# Oral presentation of histoplasmosis in non-HIV immunocompromised patient after cardiac transplant: First Brazilian case report

**DOI:** 10.1590/0037-8682-0499-2022

**Published:** 2023-01-23

**Authors:** Max Roberto Batista de Araújo, Louisy Sanches dos Santos, Lincoln de Oliveira Sant’Anna

**Affiliations:** 1 Instituto Hermes Pardini, Núcleo Técnico Operacional, Microbiologia, Vespasiano, MG, Brasil.; 2 Universidade do Estado do Rio de Janeiro, Faculdade de Ciências Médicas, Departamento de Microbiologia, Imunologia e Parasitologia, Rio de Janeiro, RJ, Brasil.

**FIGURE 1:**
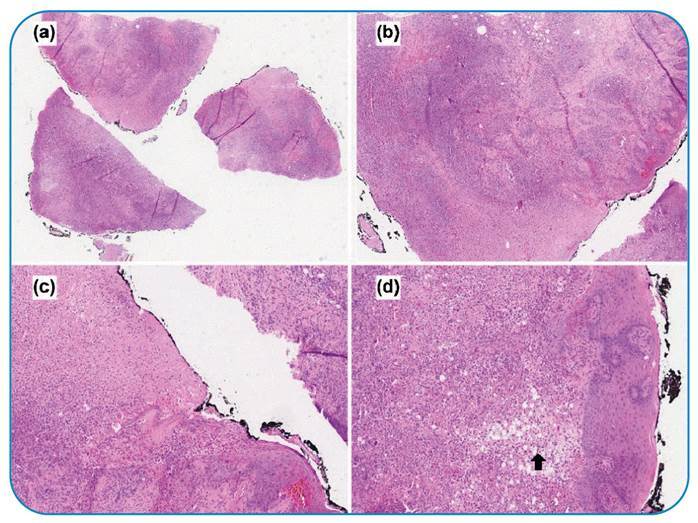
Biopsies of oral lesions and transbronchial showing granulomatous inflammation due to fungal disease. **(a)** Panoramic image of ulcerated mucosa infiltrated by a mixed inflammatory exudate (original magnification 1x-scanner: Aperio®). **(b)** Panoramic image with a focus on the ulceration (original magnification 4x-scanner: Aperio®). **(c)** Detail of the ulcer permeated by a mixed inflammatory infiltrates (original magnification 10x-scanner: Aperio®). **(d)** Clear cytoplasmic cells (black arrow) with small fungal structures, mainly in the subepithelial area (10x magnification-scanner: Aperio®).

**FIGURE 2:**
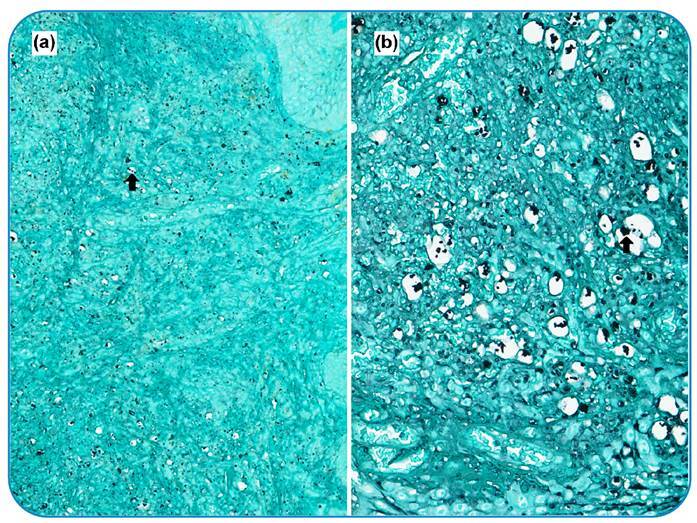
Special Gomori-Grocott staining. **(a)** Fungal structures (black arrow) in the cytoplasm of clear cytoplasmic cells (20x magnification-scanner: Aperio®). **(b)** Fungal structures (black arrow) within the cytoplasm of clear cytoplasmic cells (original magnification 40x - Scanner: Aperio®).

A 56-year-old Brazilian man with systemic arterial hypertension and type 2 diabetes mellitus was affected by ischemic heart disease that led to heart transplantation. He underwent angioplasty with stent placement and was taking immunosuppressants. Four months later, he was readmitted to the intensive care unit with an oral ulcer, fever, and holocranial headache. The biopsies of the oral lesions (Figure 1) and transbronchial showed granulomatous inflammation (Figure 2).

**FIGURE 3:**
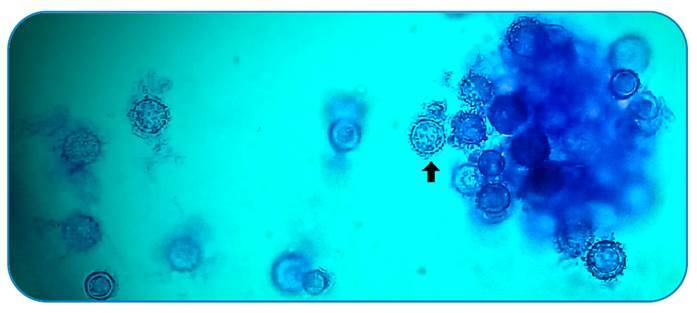
Fungal structures of the isolate were obtained from microculture on cornmeal agar at room temperature. Micromorphology shows many macroconidia (black arrow). Observation by optical microscopy at 400x magnification using cotton blue stain.

Laboratory tests yielded negative results for HIV, hepatitis virus, tuberculosis, toxoplasmosis, Cytomegalovirus, *Cryptococcus* spp. and *Paracoccidioides* spp. Blood and oral fragment samples were collected simultaneously with the application of amphotericin B lipid complex for fungal cultures and showed growth after approximately 27 days. The microculture of white colonies suggested Histoplasma spp. (Figure 3), a diagnosis confirmed by immunodiffusion tests and mass spectrometry by MALDI-TOF. This treatment was then switched to itraconazole. The patient showed clinical improvement and was discharged after 37 days of hospitalization.

Histoplasmosis is acquired mainly by the inhalation of fungi and occurs in both immunocompromised and immunocompetent individuals. Human-to-human transmission is rare and is reported only in the context of organ transplantation. Once the fungus has invaded the host, it evades immune defenses, finds a niche for its growth and reproduction, and may spread or develop a latent state within granulomas^1^. Oral manifestations in the disseminated form are particularly associated with HIV infection^2^ ^,^ ^3^. In conclusion, oral involvement is extremely rare in immunocompetent or non-HIV-infected patients, confirming that the case reported here is rare.
